# SlowMo therapy, a new digital blended therapy for fear of harm from others: An account of therapy personalisation within a targeted intervention

**DOI:** 10.1111/papt.12377

**Published:** 2022-01-12

**Authors:** Thomas Ward, Amy Hardy, Rebecca Holm, Nicola Collett, Mar Rus‐Calafell, Catarina Sacadura, Alison McGourty, Claire Vella, Anna East, Michaela Rea, Helen Harding, Richard Emsley, Kathryn Greenwood, Daniel Freeman, David Fowler, Elizabeth Kuipers, Paul Bebbington, Philippa Garety

**Affiliations:** ^1^ Department of Psychology Institute of Psychiatry, Psychology and Neuroscience King’s College London London UK; ^2^ South London and Maudsley NHS Foundation Trust London UK; ^3^ Department of Psychiatry Oxford University Oxford UK; ^4^ Oxford Health NHS Foundation Trust Oxford UK; ^5^ Oxford Institute of Clinical Psychology Training and Research Oxford University Oxford UK; ^6^ Mental Health Research and Treatment Center Faculty of Psychology Ruhr‐Universität Bochum Bochum Germany; ^7^ Sussex Partnership NHS Foundation Trust Worthing UK; ^8^ School of Psychology University of Sussex Brighton UK; ^9^ Department of Biostatistics and Health Informatics Institute of Psychiatry, Psychology and Neuroscience King’s College London London UK; ^10^ Division of Psychiatry University College London London UK

**Keywords:** blended therapy, causal interventionism, cognitive behavioural therapy, digital, human‐centred design, paranoia, psychosis, user‐centred design

## Abstract

**Objectives:**

SlowMo therapy is a pioneering blended digital therapy for paranoia, augmenting face‐to‐face therapy with an interactive ‘webapp’ and a mobile app. A recent large‐scale trial demonstrated small–moderate effects on paranoia alongside improvements in self‐esteem, worry, well‐being and quality of life. This paper provides a comprehensive account of therapy personalisation within this targeted approach.

**Design:**

Case examples illustrate therapy delivery and descriptive data are presented on personalised thought content.

**Method:**

Thought content was extracted from the webapp (*n* = 140 participants) and coded using newly devised categories: Worries: (1) Persecutory, (2) Negative social evaluation, (3) Negative self‐concept, (4) Loss/life stresses, (5) Sensory‐perceptual experiences and (6) Health anxieties. Safer thoughts: (1) Safer alternative (specific alternatives to worries), (2) Second‐wave (generalised) coping, (3) Positive self‐concept, (4) Positive activities and (5) Third‐wave (mindfulness‐based) coping. Data on therapy fidelity are also presented.

**Results:**

Worries: ‘Persecutory’ (92.9% of people) and ‘Negative social evaluation’ (74.3%) were most common. ‘General worries/ life stresses’ (31.4%) and ‘Negative self‐concept’ (22.1%) were present in a significant minority; ‘Health anxieties’ (10%) and ‘Sensory‐perceptual’ (10%) were less common. Safer thoughts: ‘Second‐wave (general) coping’ (85%), ‘Safer alternatives’ (76.4%), ‘Positive self‐concept’ (65.7%) and ‘Positive activities’ (64.3%) were common with ‘Third‐wave’ (mindfulness) coping observed for 30%. Fidelity: Only three therapy withdrawals were therapy related. Session adherence was excellent (mean = 15.2/16; *SD* = 0.9). Behavioural work was conducted with 71% of people (119/168).

**Conclusion:**

SlowMo therapy delivers a targeted yet personalised approach. Potential mechanisms of action extend beyond reasoning. Implications for cognitive models of paranoia and causal interventionist approaches are discussed.

**Practitioner points**

- Targeting causal processes such as reasoning (fast thinking) can improve paranoia and overall well‐being.
- Blended approaches, combining face therapy sessions with interactive digital content, can deliver personalised therapy across a diverse range of service users.
- Out‐of‐clinic work augmented by mobile technology can promote generalisation to daily life.

## BACKGROUND

Cognitive behavioural therapy for psychosis (CBTp) is an evidence‐based, theory‐driven approach and is recommended as a frontline treatment by the National Institute for Health and Care Excellence (National Institute for Health and Care Excellent [NICE], 2014). However, recent meta‐analyses indicate typical effect sizes in the small to moderate range (Bighelli et al., 2018; Turner et al., 2020) with evidence of significant barriers to implementation (Colling et al., 2017; Freeman et al., 2013; Goldsmith et al., 2015; Haddock et al., 2014). Improvements in effectiveness, engagement and adherence are viewed as crucial in developing psychological interventions which deliver benefit to diverse service‐users (Hardy et al., 2018).

Causal interventionist approaches aim to improve effectiveness by developing tailored interventions to target specific mechanisms that research has shown to play a causal role in the problem to be treated (Freeman, 2016; Kendler & Campbell, 2009; Mehl et al., 2015). For example, targeting of worry has been shown to mediate significant and sustained improvements in persecutory beliefs even in the context of a brief intervention (Freeman et al., 2015). Reasoning biases represent another putative causal factor and potential treatment target (Andreou et al., 2017; Freeman & Garety, 2014). Adopting a dual‐process model of reasoning (Epstein, 1994; Evans & Stanovich, 2013; Kahneman, 2011), SlowMo therapy has been developed as a targeted (causal interventionist) approach which aims to build awareness of the day to day impact of reasoning biases (i.e., Jumping to Conclusions (Dudley et al., 2016; Garety et al., 2005; So et al., 2016) and belief inflexibility (Freeman et al., 2004; Garety et al., 2005; McLean et al., 2016; Sanford et al., 2014; So et al., 2012). The hypothesised therapeutic processes of change of SlowMo are understood within the context of dual process models of cognition (e.g., Epstein et al., 1996; Evans & Stanovich, 2013; Kahneman, 2011). Specifically, SlowMo therapy aims to optimise analytic or type 2 thinking (‘slow thinking’) and build awareness of the rapid type 1 reasoning (‘fast thinking’) which is characteristic of paranoia (Ward & Garety, 2017). The mobile app represents an ‘extended mind resource’, which facilitates type 2 processing while also reducing the cognitive load through easy‐to‐access personalised content. SlowMo therapy aims are framed, for accessibility, as reducing ‘fast thinking’ and promoting use of ‘slow thinking’ (considering evidence and alternative explanations) with a focus on real‐world change.

The product of over a decade of iterative development (Garety et al., 2015; Hardy et al., 2018; Ross et al., 2011; Waller et al., 2011, 2015), SlowMo therapy is the first digital blended therapy for paranoia, employing face to face therapy sessions with interactive digital content and using mobile technology to promote generalisation to daily life. SlowMo therapy has recently been tested in a large‐scale randomised controlled trial with *N* = 362 participants (Garety et al., 2021). The overall pattern of results indicated that SlowMo was beneficial for paranoia: 10 /11 paranoia measures at 12 weeks and 8/11 at 24 weeks demonstrated significant small–moderate effects, and sustained moderate effects were observed on all observer‐rated measures of persecutory delusions, with a shorter therapy duration compared to traditional CBTp, which involves a minimum of 16 sessions over 6 months. Important improvements in self‐esteem, worry, well‐being and quality of life were also reported. Consistent with the theory‐driven design and treatment rationale, improvements in slower thinking (acknowledging the possibility of being mistaken) were found to mediate change in paranoia at 12‐ and 24‐week follow‐ups. However contrary to hypothesis, reduced JTC (fast thinking) did not mediate change in paranoia while worry, which was not directly targeted, was a significant mediator. These findings suggest that the potential therapeutic mechanisms of action of SlowMo therapy and utility of the digital tools, may extend beyond reasoning biases. To date, SlowMo therapy has been described in terms of its human‐centred design approach and treatment rationale. A detailed account of personalised therapy delivery is now required for building an understanding of how this novel blended approach facilitates positive change, and to inform further developments for real‐world implementation.

### Aim

This paper provides a comprehensive account of SlowMo therapy as delivered in the recent large‐scale trial (Garety et al., 2021), with a focus on illustrating personalised therapy delivery. New data on therapy fidelity, including session adherence and behavioural work adherence, are also presented.

## METHOD

### SlowMo therapy delivery – structure, personalisation and therapeutic targets

Core aspects of SlowMo therapy are described with examples (purposively selected to illustrate common responses to therapy from a pool of individuals who have given consent to use quotes). Personalised thought content from the SlowMo webapp was coded (by RH) using a novel system of categorisation, developed by RH, AH and TW. The sample (*n* = 140 participants) comprised all individuals randomised to receive SlowMo therapy for whom system analytics adherence data were collected (some data were missing due to a coding bug identified early in the trial). Development involved an initial scoping of thought content against *a priori* SlowMo treatment targets and established mechanisms of change within psychological interventions for psychosis. The emergent categories (and associated definitions) were subject to iterative review and refinement resulting in the following final categories (see Results for full definitions and illustrative examples).

#### Worries

- Persecutory – Fear of intentional harm from others, ideas of reference and thought broadcasting.
- Negative social evaluation – Judgement from others where no intention to harm is stated or implied.
- Loss/life stresses – Stress, negative emotions (unspecified or general cause), catastrophic or intrusive thoughts, fear of harming others.
- Negative self‐concept – Negative self‐evaluative statements.
- Sensory‐perceptual experiences – Derealisation, hallucinations and dreams.
- Health anxieties – Worry about own mental or physical health, including worry about self‐harm.

#### Safer thoughts

- Safer alternative – specific alternatives to distressing thoughts.
- ‘Second‐wave’ coping – generalised coping statements, attentional or practical strategies, normalisation, general advice.
- Positive self‐concept – Positive self‐evaluative statements.
- Positive activities – Soothing, enjoyable or rewarding activities.
- ‘Third‐wave’ coping – mindfulness, acceptance and compassion‐focussed.

Data are presented with respect to (i) the number (and percentage) of people recording thought content within each category and (ii) total number of thought ‘bubbles’ recorded in each category (and percentage of total worry or safer thoughts). To assess inter‐rater reliability a randomly selected subsample of participants (10%; *n* = 14) was independently rated by TW.

### Therapy fidelity – engagement, session adherence and behavioural work adherence

Building on previously reported therapy completion and webapp fidelity (Garety et al., 2021), we present new data, drawn from standardised trial therapy records of individuals randomised to receive therapy (*n* = 181) on the following:

- Therapy engagement and withdrawals – Including relatedness to therapy (Related; Possibly related; Unrelated) with contextual information on reason for therapy‐related withdrawal.
- Session adherence – Targeting of therapeutic processes pre‐specified in the clinical protocol (eight processes per module), for example, ‘Delivery of normalising message’; ‘Focus on worries about harm from others’; ‘Use of vignettes to allow person to reflect on their experiences’) was self‐rated by therapists after each module (0 = “Not at all delivered”, 1 = “Partially delivered”, 2 = “Successfully delivered”).
- Behavioural work adherence – Percentage of people for whom out of clinic work was a feature of therapy and mean number of times.

## RESULTS

Demographics for the 181 people randomised to receive SlowMo therapy are shown in Table 1.

**TABLE 1:** Baseline characteristics of the individuals randomised to receive SlowMo therapy

	SlowMo therapy (*n* = 181)
Age^a^	43.1 (11.7)
Sex	
Male	132 (72.9)
Female	49 (27.1)
Self‐defined ethnicity	
White	120 (66.3)
Black Caribbean	9 (5.0)
Black African	12 (6.6)
Black Other	16 (8.8)
Indian	0 (0.0)
Pakistani	4 (2.2)
Chinese	1 (0.6)
Other	19 (10.5)
Owning a smartphone?	
Yes	122/168 (77.2)
No	46/168 (22.8)
Site	
London	66 (36.5)
Oxford	49 (27.1)
Sussex	66 (36.5)
Diagnosis	
Schizophrenia	116 (64.1)
Schizoaffective	30 (16.6)
Delusional Disorder	3 (1.7)
Psychosis (other)	32 (17.7)
Time in contact with services	
<1 year	7 (3.9)
1–5 years	22 (12.2)
6–10	40 (22.1)
11–20	70 (38.7)
20+	42 (23.2)

Data are *n* (%) or ^a^mean (*SD*).

### Therapy team, training, and supervision

A total of 11 therapists (Clinical Psychologists *N* = 10, Counselling Psychologists *N* = 1) delivered therapy across the 3 sites (London, Oxford, and Sussex). All were experienced clinicians with expertise in cognitive behavioural therapy for psychosis. The team included six main trial therapists, delivering the majority of therapy and a further five local therapists (allocated between one to three people each) either working in local recruiting teams (*n* = 3) or working across other psychosis trials within main trial sites (*n* = 2). The modular webapp structure allied to a comprehensive clinical protocol facilitated therapist training. This involved an initial orientation to the webapp and protocol (identifying module‐by‐module targeted processes and associated troubleshooting), followed by one to two supervised training cases. Weekly group supervision, including use of live audio recordings, was provided by the trial therapy lead (TW) to main trial therapists (with regular consultation provided by PG and EK); supervisory arrangements for local trial therapists was provided by site therapy leads.

### SlowMo therapy delivery – structure, personalisation and therapeutic targets

#### Therapy structure

**FIGURE 1:**
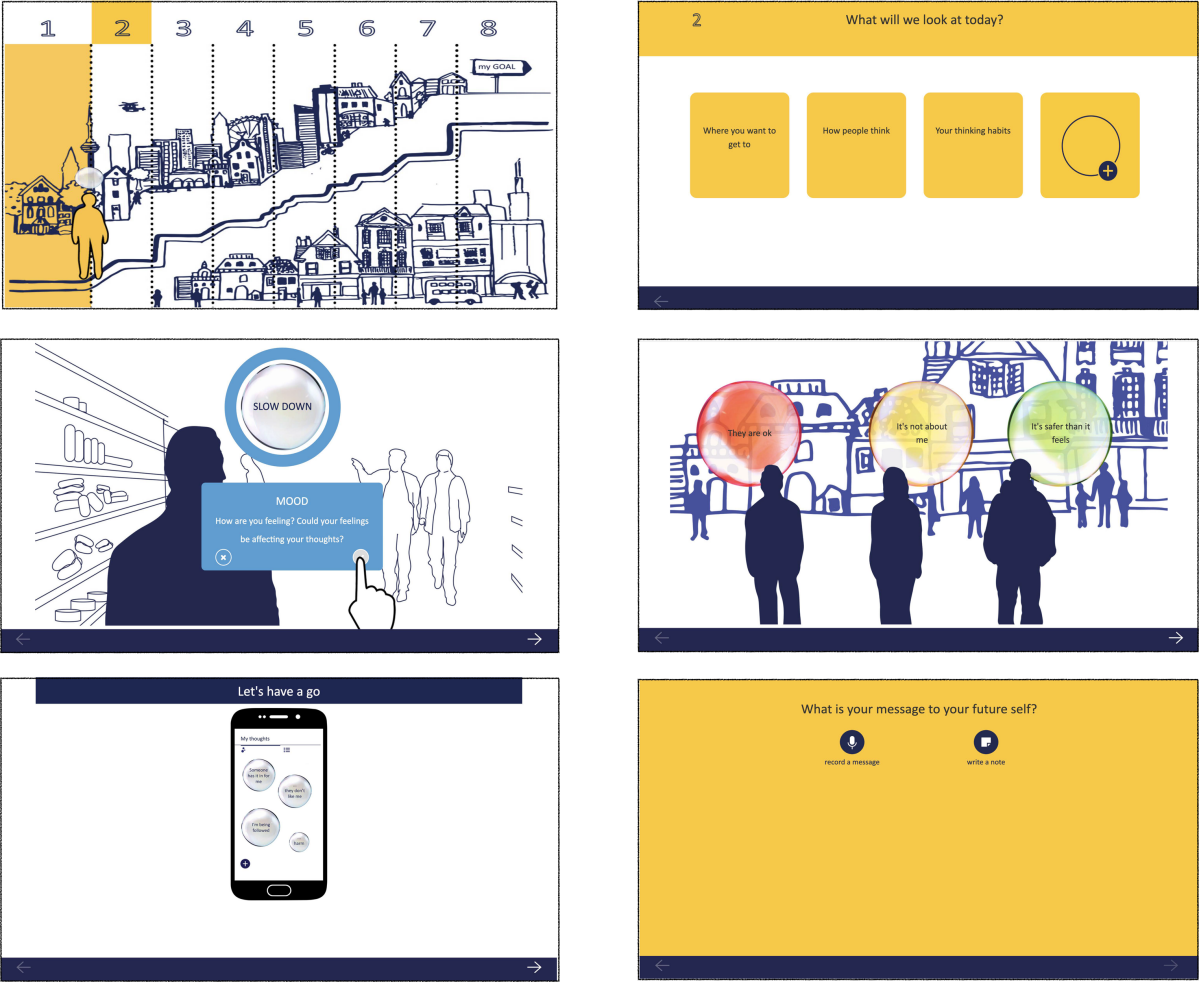
Key features of the SlowMo webapp – the journey navigation screen (top left), module agendas (top right), information and interactive features (middle panels), practice with the mobile app (bottom left) and recorded personalised messages (bottom right)

SlowMo therapy consisted of eight individual webapp modules comprising protocolised therapy components, delivered in face‐to‐face sessions typically lasting 60–90 min, delivered by trained therapists. Therapy was flexible, with sessions added, where helpful, including a pre‐therapy orientation to the SlowMo approach, splitting or combining of webapp content across meetings (tailored to individual preference and engagement) and out‐of‐clinic behavioural work to promote app use and real‐world generalisation. Therapy took place in NHS clinics, or at the home of participants, supported by the SlowMo webapp (delivered via a touchscreen laptop) – see Figure 1.

**FIGURE 2:**
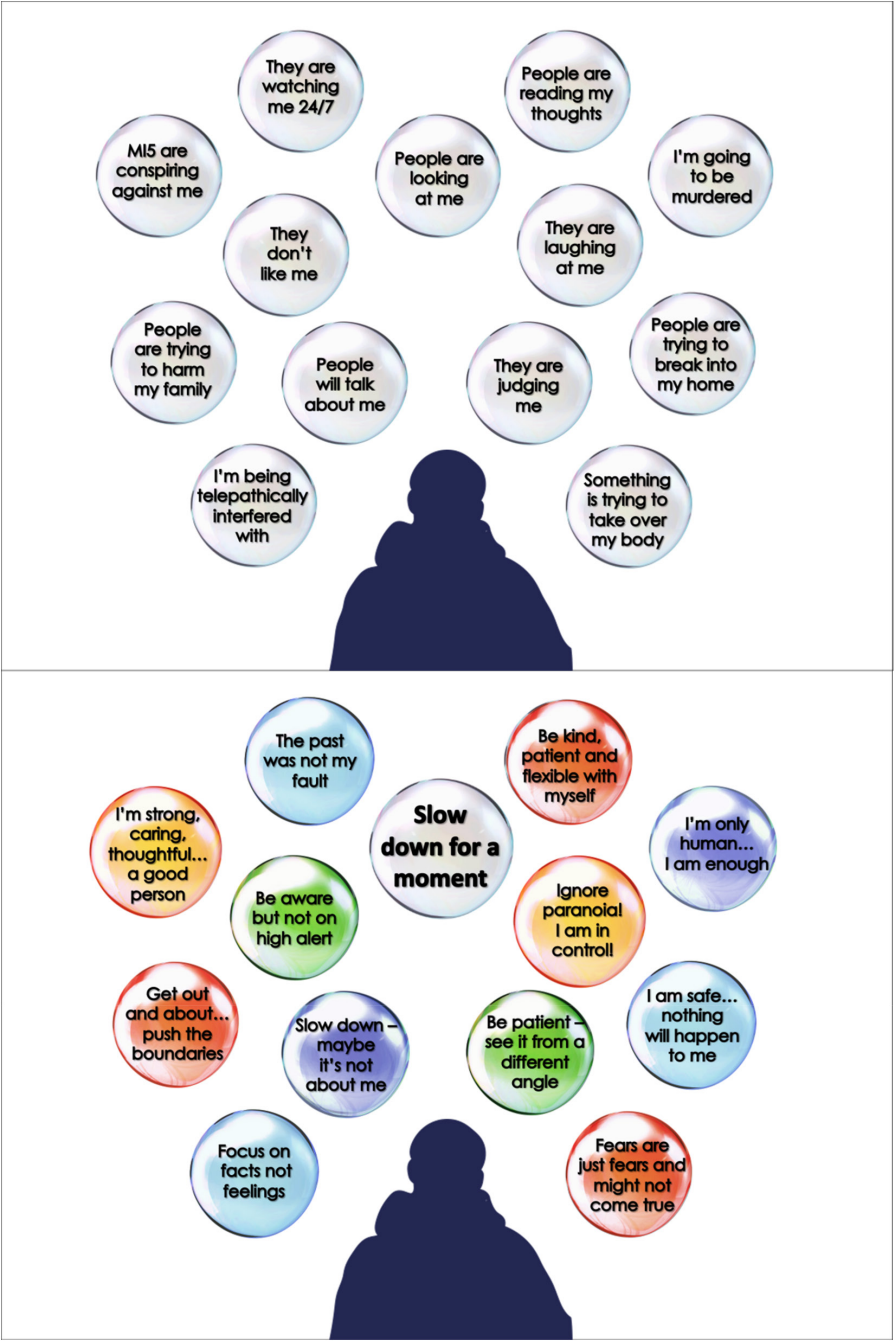
Typical worry bubbles [top] and safer alternative bubbles [bottom]

Early sessions involved building the meta‐cognitive skill of noticing thoughts and thinking habits, specifically fast and slow thinking. Thoughts are represented as bubbles, with different speeds, sizes, and transparencies, to reflect thinking habits, distress and conviction. SlowMo therapy aimed to shrink large, fast‐spinning grey bubbles and grow colourful, slow‐spinning safer thoughts (see Figure 2 for visualisation of worry and safer thought bubbles).

Each module could be undertaken in just one session or delivered over more than one session (typically 2) according to need and participant preference. All webapp modules included interactive features, providing information (through narration or recurring vignettes), games and personalised content. Personalised messages recorded at the end of each session provided a check on the delivery of intended learning (see Table 2). In Module 1, participants created personalised worry bubbles (visualised as interactive spinning bubbles) and started to build colourful, engaging safer thought bubbles, designed to promote engagement. A commonly observed Module 1 personalised message was the potentially powerful reflection ‘I’m not the only one who has these worries’. In Module 2, people learned that fast thinking is part of human nature and can be useful at times. However, fast thinking can fuel worries and thinking slowly can help in dealing with difficult situations and fears about other people. This normalising message was reinforced by the therapist sharing examples of their own fast thinking. The benefits of learning to ‘slow down for a moment’ were highlighted in Module 3 and acted as a guiding principle throughout the remaining modules. In Module 4 people practised identifying ‘safer thoughts’ as part of slowing down (e.g,. SlowMo Tips: *Could this be a coincidence? Could this be about the other person and not about me?*). Module 5 focused on understanding the impact of mood (e.g., SlowMo Tips: *Is my mood affecting the worries? How would I see things if I were in a better mood?*) and considering ways of feeling safer in daily life (e.g., shifting focus of attention and using helpful coping strategies). Module 6 outlined the potential role of past experiences in worries about others (e.g., SlowMo Tip: *The past does not predict the future*). Module 7 introduced the confirmation bias and practised how to ‘Pop the worry’ by noticing new information and identifying alternative, less distressing explanations (e.g., SlowMo tip: *Are there any clues that don't fit my fear?*). Module 8 involved a review of the therapy (including the personalised ‘messages to self’ recorded at the end of each module) in order to consolidate the habit of slowing down (Table 2).

**TABLE 2:** Summary of module learning aims and examples of personalised messages recorded in session, accessible out of session on the SlowMo mobile app

	Learning aims	Examples of personalised messages
Module 1	Normalisation of upsetting thoughts. Discuss what triggers upsetting thoughts. Introduce idea of recording (i.e., noticing) upsetting thoughts and their impact	‘Many people worry about others‐ I am not alone’ ‘There is nothing wrong with me. I am normal’
Module 2	Introduce and normalise concept of fast and slow thinking Link fast thinking with increased worry (introduce idea that slow thinking could help) Encourage reflection on own thinking habits	‘Slow down ‐ have a second thought (nothing is final)’ ‘Don't jump to conclusions‐ evaluate situation and judge whether thought is helpful’
Module 3	Practice slowing down in session with interactive games Notice a pull towards fast thinking – consider the negatives of ‘jumping to a conclusion’ Practice slowing down – noticing the impact	‘Be like a judge‐ think and make decisions slowly’ ‘It may not be what it seemed’
Module 4	Discuss slowing down including potential benefits Practice slowing down using SlowMo tips Practice generating safer thoughts	‘Slow down ‐ Don't assume too quickly. Think of other angles.’ ‘The other person might be struggling in ways I don't see’
Module 5	Discuss how mood can affect thoughts Notice if mood is affecting the person's thoughts Generate ideas for feeling safer	‘Focus on facts not feelings’ ‘Slow down, breathe, shift your focus from yourself to the surroundings’
Module 6	Discuss how the past can affect thoughts Practice noticing if the past is affecting the person's thoughts Practice generating safer thoughts	‘I’ve struggled in the past but I’m using my experiences to help change my life’ ‘The past is many thousands of miles away. It isn't about me now’.
Module 7	Discuss how we can get stuck in ways of seeing things and reacting (confirmation bias) Notice clues that things are not the way we fear (popping worry bubbles) Practice popping worries – generating safer thoughts	‘Slow down ‐ could my past be making me assume the worst in people?’ ‘Shift perspective ‐ zoom out to see a bigger picture’
Module 8	Reflect on progress and learning Planning for the future – choosing most helpful tips Writing a final message to future self	‘Beware the rabbit hole of over‐analysis ‐ it is a trap which separates me from others’ ‘Once you pop the bubble, you can see the situation clearly and know the context fully.’

**FIGURE 3:**
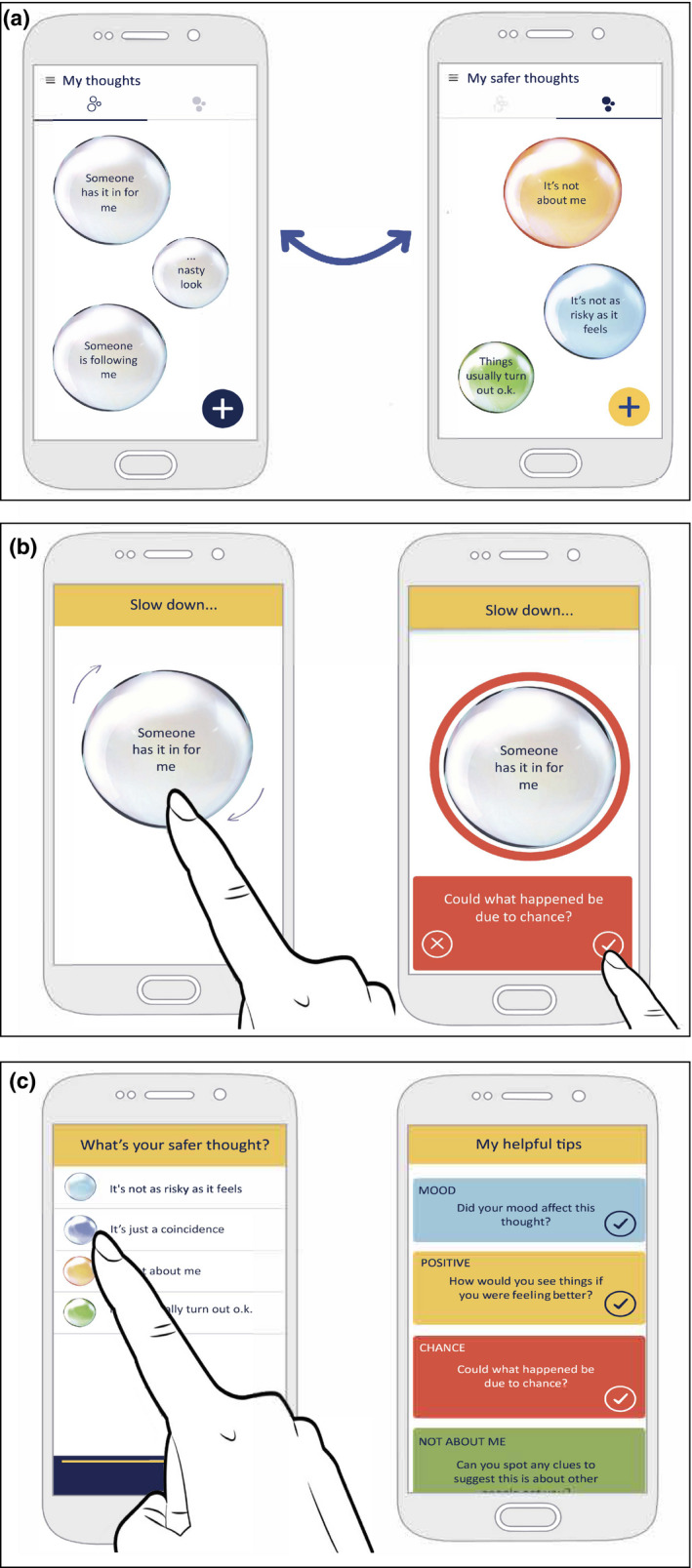
Key features of the SlowMo mobile app: worry and safer thought bubbles (top); interactive slowing down features (middle); personalised messages and SlowMo tips (bottom)

Personalised session content from each module was synchronised with a native SlowMo mobile app to promote therapy generalisation into daily life. This allowed people to notice their fears and thinking habits and interact with tailored safer alternative bubbles and SlowMo strategies, thereby finding alternative ways of managing distressing experiences in the flow of daily life (Hardy et al., 2018) ‐ see Figure 3.

It was commonly observed by clinician's during the weekly review, that usage of the mobile app during the week helped people to internalise the helpful messages developed during the therapy (see Alex; Box 1, Panel 1).

Box 1 **Two illustrative examples of SlowMo therapy**

**Panel** **1: Alex (A black british caribbean man in his 20s)**:

Alex came to SlowMo therapy with a strongly held distressing belief that someone from his past was following him and meant him harm. He feared that people were entering his flat, taking possessions and posing a threat to his young son. A key feature of therapy was Alex developing personalised alternative explanations which were accessible when needed, via the SlowMo app. A guiding principle (and key safer thought bubble) read ‘Slow down. Don't assume’. Alex used safety bubbles and messages to manage anxiety‐provoking triggers (e.g., recognising that screams and laughter on the street outside may just be ‘people having fun’). At his final session Alex's ratings of distress, conviction and fast thinking were significantly reduced. He described having learnt skills to prevent ‘inklings’ of threat becoming ‘full‐blown reacting’ and was no longer seeing thoughts and worries as ‘truth’. Through frequent early use of the SlowMo app, Alex said key messages became ‘almost automatic’ leading to a natural reduction in his app usage as key principles were internalised. His final therapy message was a reminder to himself about his new approach to dealing with worries about others: ‘I slow down…I think different, act different, and feel different’.

**Panel** **2: Biz (A white british woman in her 50s)**:

Biz came to therapy with worries about being physically and emotionally harmed by others. Prominent drivers of distress included fear of social‐evaluation (‘they think I’m lazy…selfish…a fraud’) and high levels of self‐criticism (‘I am awful. I have done something terrible’). In addition to tackling fast thinking, a major focus involved targeting negative beliefs about the self and related appraisals of self‐efficacy which contributed to Biz's worries about others. The overarching SlowMo principle (Slow down for a moment) supported Biz to approach her thoughts in a different way, whether directed towards others or inwards to herself. During sessions exploring the influence of the past, Biz discussed a core belief ‘I am worthless’ in the context of experiences of emotional neglect, bullying and social exclusion. Biz used the SlowMo principles to break cycles of fast negative thinking, low mood and avoidance. The FlowMo (focus on slower breathing) app function helped when strong affect made it difficult to ‘Slow down‐ put on my new glasses‐ see the colours!’. Biz regularly listened back to powerful messages she had audio‐recorded and interacted with colourful bubbles promoting self‐acceptance: ‘I’m not to blame‐ I’m harder on myself than anyone else’; ‘Just as I am’. Following the end of therapy Biz continued accessing her personalised SlowMo app to practice self‐acceptance and manage worries about others.

#### Personalisation within SlowMo therapy

The skill of noticing fast thinking to facilitate thought‐specific slowing down was promoted throughout. Content was individually tailored as people interacted with personalised worry bubbles, safer (positive) alternative bubbles, key learning and messages for the week ahead (recorded in text or audio form) proving engaging and useful for people:

##### John (21)

It was definitely more interactive for me, because I was actually putting input in the computer, and with the phone as well. Like when the worry was small the bubble was small and when the worry was big the bubble was bigger so you could visualise it easier and see what you needed to work on. Sometimes in my head it can feel like there's so many worries it can get overloaded. But seeing the worry bubbles, there's like about 10 on there and only a couple that are like really worrisome, I guess. And with the helpful bubbles it's just nice to look back and see ‘I found this helpful in the past so it can help me now’… something you just forget about normally.

##### Ron (58)

[The bubbles] were very, very helpful. They helped me with my thoughts… Touching the bubbles, being able to work out my thoughts… I found it really helpful. I am not, as they say, computer literate… it was a new experience for me but helped tremendously using a computer. it was very easy to use, the way it was explained…. It was perfect for me’.

##### Sophie (28)

Bubbles were eye‐catching and made therapy fun… helpful bubbles taught me that I can end the cycle of worry. The session summaries were very useful…. The tips about slowing down were brilliant‐ I learnt that I did not need to think speedily and start panicking.

### The SlowMo approach to formulation

**FIGURE 4:**
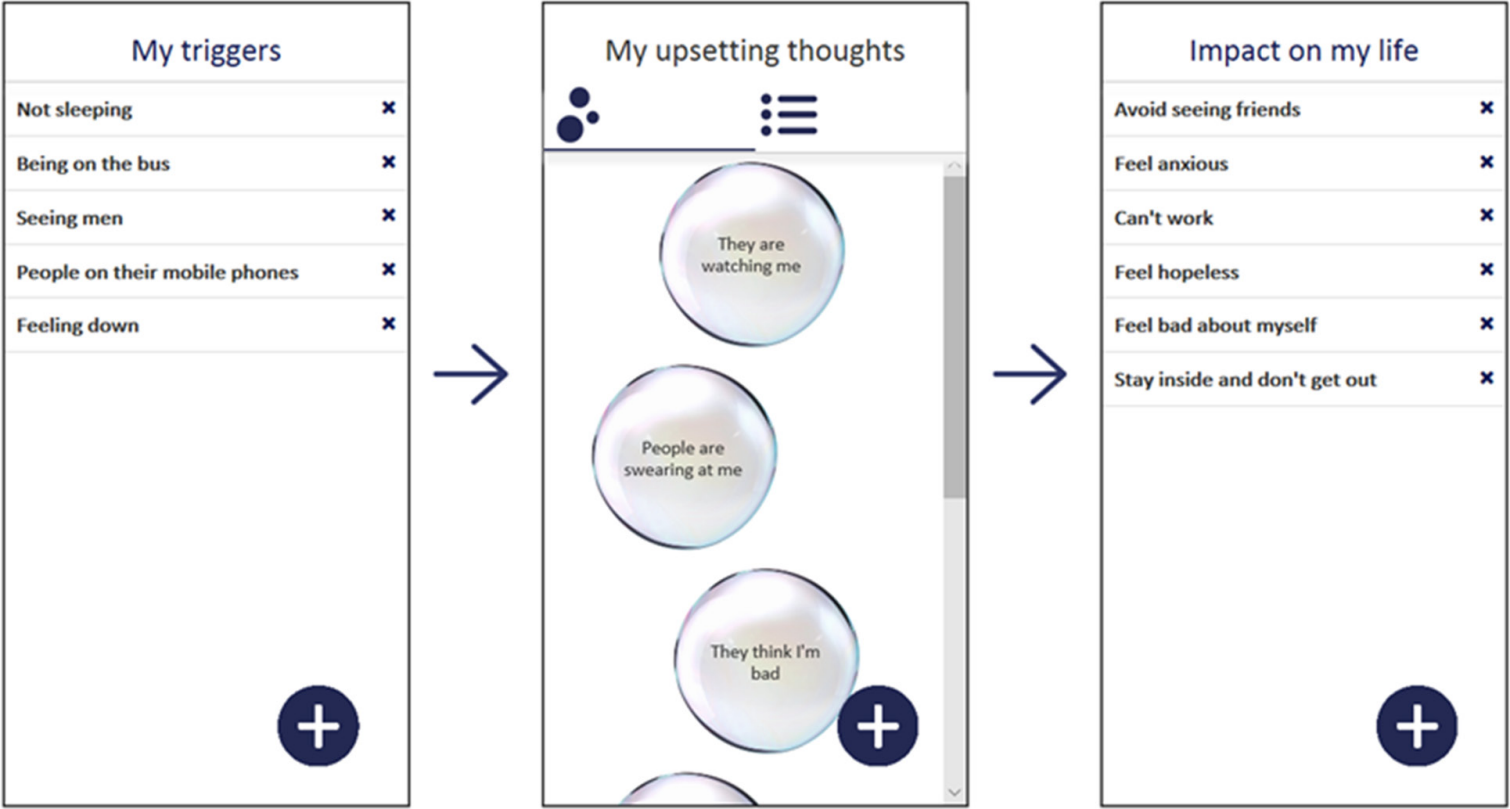
SlowMo webapp formulation

SlowMo therapy is a targeted formulation‐driven intervention and an example of a brief high intensity CBT, employing the conceptualisation of Shafran et al. (2021). A flexible approach to formulation ensured that the targeted causal processes (fast and slow thinking) were understood in a way that was individualised and accessible for the person and considered experiential as well as cognitive aspects of paranoia (Feyaerts et al., 2021). Interactions between experiences, thoughts, feelings and behaviour (Freeman, 2016; Freeman et al., 2002; Garety et al., 2001) were discussed throughout therapy, facilitated by the person's engagement with webapp content. The webapp formulation was presented using a simple ABC format (My triggers; My upsetting thoughts; Impact on my life) to promote accessibility (Figure 4). This core webapp component was revisited at the start of each session (following a weekly review and ratings of distress, conviction and fast thinking). New content was added using the touchscreen ‘+’ function (mirroring the SlowMo mobile app) allowing consolidation of out‐of‐session learning (thought bubbles were synched directly from the phone.) This formulation acted as a touchstone, facilitating reflection on triggers, fast thinking and attempts to ‘slow down’ during the week. The safer thoughts screen, which followed the formulation screen within the webapp, ensured a consistent focus on building positive, safer alternatives. For some, the formulation identified processes relating to voice‐hearing, social anxiety, low mood, inactivity, rumination, self‐criticism etc. Where helpful, additional microformulations (e.g,. highlighting reciprocal interactions between thoughts, mood and behaviour) were drawn out and discussed outside of the webapp. Voice‐hearing was addressed by respecting the person's experiences and exploring ways in which fast and slow thinking might exacerbate or ameliorate the associated distress, in order to develop safer, alternative thoughts about voices and their impact.

Three recurring vignettes (Sam, Nadia and James) featured across sessions and were identified as a helpful aspect of therapy by both participants and therapists. Interaction with vignettes allowed people to decentre from their own worries, reducing the potential ‘stuckness’ that can be a feature of working with strongly held beliefs. People valued the opportunity to identify the fast thinking of others and advise on how they could slow down and manage situations:

#### Ron (58)

It was helpful because the experiences they [Sam, Nadia and James] were giving me were similar to the experiences I felt myself. It was just nice to listen to people like that, who got similar problems and that helped me tremendously to get through.

#### John (21)

Thinking slow and thinking fast may seem like obvious to some people but you don't always remember to do that. Hearing other people's experiences was helpful because in the beginning I felt really alone, I felt it was just me thinking like this. The examples weren't exactly what I was experiencing but it was just nice to hear other people's points of view.

Processes relevant to the person's own experiences and formulation could then be broached in a nonchallenging manner. In addition to a consistent focus on ‘in the moment’ processing of threat, including the role of attention and use of safety behaviours, SlowMo therapy contextualises fear of harm from others within the broader context of the person's life (including social relationships and associated schema or ‘internal working models’). For some this developmental perspective involved sharing a diagrammatic developmental formulation (see Biz, Box 1, Panel 2 for an example where the influence of past experiences was particularly prominent). During Module 6, Sam, Nadia and James discuss the impact of mistreatment, bullying and exclusion on their current lives. Crucially, in the context of past trauma and adverse experiences, use of vignettes meant the person retained control over decisions to share personal experiences:

#### Ron (58)

I don't really know the word to describe how it made me feel to release what my past was all about. It was a coming out of a bubble into the world. SlowMo has helped me talk about my experiences more to different professionals, to my family. I talk more about it now than what I used to but it's in the past now, it doesn't haunt me so much as what it used to…It makes you feel you are part of this world, I haven't had that for a very long time. I don't want to die anymore…. I just want to live life as well as I can… It's just changed my life.

#### Integration of ‘out of clinic’ work

Practising the SlowMo principles using the mobile app in real‐world settings was introduced, from the start, as a routine and potentially helpful aspect of therapy. The individual approach remained collaborative with discussion of any obstacles to engagement. Out‐of‐clinic work was, wherever possible, linked to the person's goals for therapy, for example, practising slow thinking while using public transport as a step towards reconnecting with social activities. This work was designed to build on strengths and reflect the processes formulated, collaboratively, during therapy, with the aim of promoting therapy generalisation to the real‐world.

Biz (a white British woman in her 50s; see Box 1, Panel 2) provides an example of important out‐of‐clinic work. Biz's main worries centred on fear of assault (grounded in past experiences) together with worries about social evaluation and concerns that other people were monitoring her. Early out‐of‐clinic work involved trying out the mobile app during a short trip to the local shops with the therapist. This allowed Biz to notice worries, fast thinking and associated feelings in her daily environment (the main mobile app functions introduced in the first two modules). Additional triggers, worries and impact were identified and added to the webapp formulation screen at the start of the next session, supporting ongoing assessment and formulation. Discussion around out‐of‐clinic work and in‐session vignettes led to a collaborative formulation, which highlighted the role of threat‐focussed attention and safety‐seeking behaviours. These were framed as natural responses to past experiences, but which nevertheless fuelled Biz's current worries. A longer piece of out‐of‐clinic work (following Module 5) was conducted as a standalone session. Biz tested out dropping safety behaviours (‘avoiding eye contact’) and attempted to use the personalised SlowMo app content to shift from a threat‐focussed mindset while navigating busy streets and shops. For Biz and others on the trial, the SlowMo message of slowing down and gathering more information augmented the standard cognitive approach to behavioural work, with the phone acting as an ‘extended mind resource’ (Ward & Garety, 2017).

#### Therapeutic targets and mechanisms of change

Table 3 presents data on the novel categorisation of personalised worry and safer thought bubbles. IRR analysis indicated ‘substantial agreement’ (following (Landis & Koch, 1977)) for both distressing (kappa = .68; % agreement = 79.8%) and safer (kappa = .76; % agreement = 83.0%) thoughts. Worry bubble content encompassed the full spectrum of paranoia (Bebbington et al., 2013; Freeman et al., 2005). Consistent with the SlowMo treatment rationale, the most common distressing thought categories, were ‘Persecutory’ (observed in over 90% of people) and ‘Negative social evaluation’ (recorded in three quarters of people). However, there was clear evidence of the bubbles being used to capture a broader range of content, with a significant minority of people recording bubbles related to general ‘Life stresses’ and ‘Negative self‐content’. The most common safer bubbles reflected ‘Second‐wave coping’, recorded by 85% of the sample, followed by ‘Safer alternatives’, recorded by three quarters of the sample. However, as with the worry bubbles, the fact that two thirds of the sample recorded bubbles focused on ‘Positive self‐concept’ and ‘Positive activities’ demonstrates that engagement with digital tools extended beyond an exclusive focus on reasoning. Content reflecting self‐affirming messages (‘I’m only human, I am enough’; ‘Be kind, patient and flexible with myself’) was particularly encouraged by the therapist where the evolving formulation pointed to self‐criticism, guilt and associated low mood as factors contributing to distress (see Biz; Box 1, Panel 2).

**TABLE 3:** Definitions, illustrative examples and descriptive statistics for categories of distressing and safer thoughts showing (i) the number (%) of people recording bubbles in each category and (ii) number of bubbles recorded in each category (and as a % of total worry and safer thought bubbles)

Thought type	Category	Definitions	Illustrative example	*n* (%) people recording thoughts within category	Total *n* bubbles per category (% of all recorded thoughts)
**Distressing**				***n* = 140**	***n* = 989**
	Persecutory	Thoughts related to others’ intention to harm, ideas of reference and thought broadcasting	‘I worry that others are part of a conspiracy that is against me’, ‘People reading my thoughts’	130 (92.9)	494 (50.0)
	Negative social evaluation	Thoughts related to judgement from others where no intention to harm is stated or implied	‘They think I'm a gossip’, ‘People are judging me’	104 (74.3)	298 (30.1)
	Loss/life stresses	Life stresses, negative emotions (unspecified or general cause), catastrophic or intrusive thoughts, fear of harming others	‘I might become homeless’, ‘Uncontrollable thoughts’, ‘Scared that I'm going to crack and seriously hurt someone’	44 (31.4)	80 (8.1)
	Negative self‐concept	Negative self‐evaluative statements	‘I am unlovable’	31 (22.1)	67 (6.8)
	Sensory‐perceptual experiences	Derealisation, hallucinations and dreams	‘Am I passed out somewhere and dreaming this reality’, ‘Voices start unexpectedly’	14 (10.0)	26 (2.6)
	Health anxieties	Worry about own mental or physical health, including worry about self‐harm	‘Worrying about going crazy again and not being able to recognize it’, ‘I worry that I will hurt myself’	14 (10.0)	24 (2.4)
**Safer**				***n *= 140**	***n* = 1393**
	Second‐wave coping	Attentional strategies, practical changes, normalisation, breath retraining, general advice to self inc. to use the SlowMo app, encouragement	‘Everybody has negative and distressing thoughts at times ‐ its normal’, ‘Working to get my license back’, ‘Use FlowMo to slow my breathing’, ‘positive steps are possible’	119 (85.0)	458 (32.9)
	Safer alternative	All direct counters to individual distressing thoughts, inc. reiterations of SlowMo messages	‘No‐one said I was annoying! No evidence!’, ‘Could my feelings be affecting this thought?’, ‘Slow down and look at the facts’	107 (76.4)	348 (25.0)
	Positive self‐concept	Positive self‐evaluative statements, including linking self to religion	‘I am a kind person’, ‘Jesus loves me’	92 (65.7)	297 (21.3)
	Positive activities	Soothing, enjoyable, or rewarding activities inc. religious activities	‘Going out for a meal with friends’, ‘Gardening’, ‘Praying’	90 (64.3)	233 (16.7)
	Third‐wave coping	Self‐compassion, acceptance of thoughts, mindfulness, meditation	‘Be kind to yourself’, ‘Being the observer (letting go)’ ‘Meditation helps voices become more distant’	42 (30.0)	57 (4.1)

### Therapy fidelity – engagement, session adherence and behavioural work adherence

#### Therapy engagement and withdrawal

As previously reported (Garety et al., 2021), over 85% of individuals who attended the first session went on to complete all eight webapp modules (See Table 4) suggesting a high level of acceptability. Withdrawals, once therapy had commenced, occurred for 23 participants. Possible evidence of withdrawal specifically related to SlowMo therapy was observed in only three instances; these occurred within the first two modules with reasons relating to the focus of the intervention, concerns about technology and concerns about talking about the past.

**TABLE 4:** Data on therapy fidelity including completion, withdrawals, session adherence and behavioural work adherence

	*N*	*n*	*%*
Therapy completion?			
Total sample	181		
Yes		145	80
No		36	20
Those attending ≥1 session	168		
Yes		145	86
No		23	14
Therapy withdrawals	36		
SlowMo related? Yes		0	–
Possibly		3	–
No		33	–
Session adherence^a^			
Adherent webapp delivery	168	159	95
Therapy process (max = 16)	162	–	Mean = 15.2 (*SD* = 0.9)

	*N*	*M* (*SD*)	Range
N^o^ of face‐to‐face sessions			
Overall sample	181	7.85 (3.06)	0–15
Those attending ≥1 session	168	8.43 (2.31)	1–15
Those completing all sessions	145	9.19 (1.14)	7–15

	*N*	*n*	%
Behavioural work adherence^b^			
Sessions inc. going out?	168		
Yes		119	71
No		49	29

	*N*	*M* (*SD*)	Range
Number of times out with app?			
If ‘yes’ to out of clinic work	118 (1 missing)	2.64 (1.79)	1–9
Overall sample	167	1.86 (1.93)	0–9

^a^ Adherence defined as no more than one webapp component missed for any module.

^b^ Practice with mobile app within and between face‐to‐face sessions).

#### Session adherence

As previously reported (Garety et al., 2021), the mean number of completed webapp modules was 6.8 (*SD* = 2.6) across the therapy arm; rising to 7.3 (*SD* = 1.9) for those attending at least one session. The mean duration per module was 75 min (*SD* = 29 min), including time spent out‐of‐clinic. Fidelity in webapp delivery (no more than one webapp component missed for any module) was achieved for 95% of participants with fidelity ratings of at least 90% for each of the eight modules. With respect to new data (reported here for the first time), the mean therapy process fidelity (across all modules) was over 15 out of a maximum of 16, confirming consistent delivery of the clinical protocol.

#### Behavioural work adherence

Out‐of‐clinic work was a feature of SlowMo therapy for over 70% of those who attended therapy, taking place, on average, 1.9 times during therapy.

## DISCUSSION

SlowMo therapy has small–moderate effects on its treatment target of paranoia (fear of harm from others) together with broader benefits in self‐esteem, worry, well‐being and quality of life, as reported separately (Garety et al., 2021). For the first time this paper provides a comprehensive account of real‐world SlowMo therapy delivery, illustrating therapy personalisation within this targeted approach. Data on personalised thought bubbles show a consistent focus on the *a priori* SlowMo treatment targets (i.e., persecutory thoughts and fear of negative evaluation) with the most common helpful bubbles reflecting paranoia‐specific ‘safer thoughts’ and ‘second‐wave’ (general) coping. However, the results also demonstrate use of the digital tools to target a broader range of worries (including general life stresses) and helpful bubbles focussing on ‘positive self‐concept’ and ‘positive activities’. The blended digital approach delivered excellent therapy fidelity with a mean session adherence rating of 15.2 (out of 16) and delivery of out‐of‐clinic work for over 70% of people attending therapy. These findings, supported by the strong focus on personalisation and real‐world impact, provide encouraging evidence for future implementation. SlowMo's flexible blending of a causal interventionist approach with use of interactive digital technology, carries implications for the future development of psychological treatments.

### Delivering interventions that are targeted, flexible and personalised

The NICE‐recommended minimum of 16 sessions over 6 months of CBTp is often extended in routine practice due to complex social and psychological factors that affect therapy engagement (e.g., ongoing experiences of abuse, threat of homelessness and removal of benefits). So‐called ‘therapy drift’ (Waller & Turner, 2016) can be a natural consequence of clinical complexity, potentially leading to confusion for both the person and the clinician over the therapy aims. Briefer targeted high‐intensity interventions (Shafran et al., 2021) such as SlowMo, provide a clear structure, facilitating a consistent focus on the processes most likely to result in real‐world change. The SlowMo trial demonstrated improvements which match or exceed those typically observed for standard CBTp, delivered in a shorter number of sessions (Garety et al., 2021). The design of SlowMo therapy reflects the intimate linking of thinking ‘process’ and thought ‘content’ in real‐world contexts. Building the skill of noticing fast thinking can be viewed as a process‐level ‘default‐intervention’ on automatic processing (Ward & Garety, 2017), while personalised, accessible ‘safer’ content facilitates slower thinking in daily life. Delivering personalisation and flexibility within a coherent structure is a guiding principle of the SlowMo approach, in light of the clinical complexity noted above. The overarching principle of ‘Slowing down for a moment’ can be viewed as a foundational process of ‘stepping back’ (decentring) from unhelpful automatic thoughts, a cornerstone of cognitive therapy. As demonstrated in the personalisation data, this also allowed adaptation of the SlowMo approach to nonparanoia thought content, particularly relating to generalised worries and self‐concept, both of which are key issues in the context of psychosis (Collett et al., 2016; Freeman et al., 2014, 2021). This study, therefore, highlights the importance of ensuring that tailored content and personalised meanings should remain central to the therapy experience of targeted treatments for psychosis.

### Causal interventionism and a cascade of positive effects

Cognitive models of psychosis propose that biopsychosocial processes interact to cause and maintain paranoia (Freeman, 2016; Freeman et al., 2002; Garety et al., 2007). Application of theory to clinical practice requires a distillation of complexity in the service of collaborative formulation and intervention, for example, the commonly used ‘vicious cycles’ foregrounding relationships between thoughts, feelings and behaviour. The SlowMo trial provides encouraging evidence that targeting reasoning biases (promoting slower thinking and belief flexibility) can lead to improvement in paranoia as would be hypothesised by cognitive models of persecutory beliefs (Freeman, 2016; Freeman et al., 2002). However, the ‘dappled’ interplay of aetiological factors (Kendler, 2012), located across levels of explanation ranging from the micro (e.g., molecular) to the macro (e.g., culture) belies a reductionist interpretation of causal interventionist treatment for psychosis. Psychological processes involve dynamic variables, which are likely to interact in a context‐dependent, nonlinear, multilevel, even ‘chaotic’ manner (Sulis, 2018; Uher, 2018). While treatment effects for persecutory beliefs can be improved by targeting delineated processes, a targeted therapy such as SlowMo should nevertheless also be designed to facilitate a cascade of positive effects across interacting processes which include reasoning, worry, safety behaviours, anomalous experience and self‐concept (Freeman et al., 2021). Crucially, the personalised formulation‐led delivery of SlowMo, allied to the focus on real‐world change, ensures that the experiential aspects of paranoia are foregrounded throughout therapy (Feyaerts et al., 2021).

### Use of blended therapy and human‐centred design

SlowMo therapy is, to our knowledge, the first blended digital therapy for paranoia, using an interactive webapp synchronised to a mobile app to provide access to personalised messages and strategies during everyday life. The iterative design approach involved service users and other stakeholders at every stage (Hardy et al., 2018), and resulted in design concepts (e.g., the visualisation of thoughts as bubbles) which were well‐received throughout the trial. Crucially, the human‐centred design process (Clarkson & Coleman, 2015; Norman, 2013) purposively sampled individuals at the ‘extremes’ of normal distributions relating to cognitive difficulties, prior technology experience and attitudes to therapy, who are often excluded from participatory co‐design approaches (Hardy et al., 2018). The high rates of therapy completion point to an intervention which is engaging and accessible across a diverse range of service users. The excellent therapy fidelity demonstrates consistent delivery of the therapy protocol across participants with varying degrees of digital literacy, some of whom had never used a smartphone (Hardy et al., (Unpublished Data)). Interactive digital features allowed tailoring and pacing of delivery to the individual. Recurring vignettes were identified as particularly helpful in promoting a normalising message and opening clinical discussions in a naturalistic manner. The SlowMo trial, therefore, provides evidence for the potential of human‐centred design to improve the acceptability, usability and engagement of psychological interventions for psychosis, addressing the needs of those for whom standard CBTp delivery presents barriers to engagement (Hardy et al., (Unpublished Data)).

It should be noted that the service‐user quotes presented in the current paper have been purposively selected to illustrate core features of SlowMo therapy. A forthcoming service‐user‐led qualitative study represents a coproduced investigation of the participant experience of receiving SlowMo therapy, which will be particularly informative in the ongoing development of the intervention. The session adherence data presented here were based on therapist self‐report using standardised therapy booklets. While the structured nature of SlowMo meant that rating of delivery of each webapp component was straightforward, a more fine‐grained analysis of the webapp analytics (e.g., duration spent on each component) would be informative for future implementation studies.

### Future directions

SlowMo therapy has been shown to be effective in its aim of improving paranoia, delivering meaningful change in a relatively brief duration of therapy. Important learning from tailoring the approach across diverse presentations will be carried into the next iteration including further improvements to the flexibility and usability of the digital platform. The fact that people adapted their personalised ‘slower safer thought bubbles’ to include positive self‐related content, together with the observed improvements in self‐esteem, worry, well‐being and quality of life, suggest the potential for transdiagnostic application.

This paper has provided a comprehensive account of SlowMo therapy as delivered in a recent large‐scale randomised trial alongside encouraging evidence on therapy personalisation and fidelity. Future work will focus on the development and implementation of the SlowMo approach addressing the clinical and technical challenges of delivery in routine NHS services. SlowMo therapy represents a forerunner of what could become a new generation of targeted blended treatments which harness digital technology and human‐centred design to improve effectiveness, accessibility and engagement of psychological therapy for psychosis.

## CONFLICTS OF INTEREST

Drs Garety, Emsley, Greenwood, Freeman, Fowler, Kuipers, Bebbington and Hardy reported receiving grants from the National Institute of Health Research (NIHR) during the conduct of the SlowMo trial. Dr Freeman declared personal fees from University of Oxford spin‐out company, Oxford VR., outside the submitted work. No other disclosures were reported.

## AUTHOR CONTRIBUTION

**Thomas Ward:** Conceptualization (equal); Data curation (equal); Formal analysis (equal); Investigation (equal); Methodology (equal); Project administration (equal); Resources (equal); Supervision (equal); Visualization (equal); Writing – original draft (equal); Writing – review & editing (equal). **Amy Hardy:** Conceptualization (equal); Data curation (equal); Formal analysis (equal); Funding acquisition (equal); Investigation (equal); Methodology (equal); Project administration (equal); Resources (equal); Supervision (equal); Visualization (equal); Writing – original draft (equal); Writing – review & editing (equal). **Rebecca Holm:** Conceptualization (equal); Data curation (equal); Formal analysis (equal); Methodology (equal); Visualization (equal); Writing – review & editing (equal). **Nicola Collett:** Conceptualization (equal); Data curation (equal); Formal analysis (equal); Investigation (equal); Methodology (equal); Project administration (equal); Supervision (equal); Visualization (equal); Writing – original draft (equal); Writing – review & editing (equal). **Mar Rus‐Calafell:** Conceptualization (equal); Data curation (equal); Formal analysis (equal); Investigation (equal); Methodology (equal); Project administration (equal); Resources (equal); Supervision (equal); Visualization (equal); Writing – original draft (equal); Writing – review & editing (equal). **Catarina Sacadura:** Conceptualization (equal); Data curation (equal); Formal analysis (equal); Investigation (equal); Methodology (equal); Project administration (equal); Supervision (equal); Visualization (equal); Writing – original draft (equal); Writing – review & editing (equal). **Alison McGourty:** Conceptualization (equal); Data curation (equal); Investigation (equal); Methodology (equal); Supervision (equal); Writing – original draft (equal); Writing – review & editing (equal). **Claire Vella:** Conceptualization (equal); Data curation (equal); Formal analysis (equal); Investigation (equal); Methodology (equal); Project administration (equal); Visualization (equal); Writing – original draft (equal); Writing – review & editing (equal). **Anna East:** Conceptualization (equal); Data curation (equal); Formal analysis (equal); Investigation (equal); Methodology (equal); Project administration (equal); Visualization (equal); Writing – original draft (equal); Writing – review & editing (equal). **Michaela Rea:** Conceptualization (equal); Data curation (equal); Formal analysis (equal); Investigation (equal); Methodology (equal); Project administration (equal); Visualization (equal); Writing – original draft (equal); Writing – review & editing (equal). **Helen Harding:** Conceptualization (equal); Funding acquisition (equal); Investigation (equal); Methodology (equal); Writing – original draft (equal); Writing – review & editing (equal). **Richard Emsley:** Conceptualization (equal); Funding acquisition (equal); Methodology (equal); Writing – original draft (equal); Writing – review & editing (equal). **Kathryn Greenwood:** Conceptualization (equal); Funding acquisition (equal); Investigation (equal); Methodology (equal); Project administration (equal); Supervision (equal); Writing – original draft (equal); Writing – review & editing (equal). **Daniel Freeman:** Conceptualization (equal); Funding acquisition (equal); Investigation (equal); Methodology (equal); Project administration (equal); Supervision (equal); Writing – original draft (equal); Writing – review & editing (equal). **David Fowler:** Conceptualization (equal); Funding acquisition (equal); Investigation (equal); Methodology (equal); Project administration (equal); Supervision (equal); Writing – original draft (equal); Writing – review & editing (equal). **Elizabeth Kuipers:** Conceptualization (equal); Funding acquisition (equal); Investigation (equal); Methodology (equal); Project administration (equal); Supervision (equal); Writing – original draft (equal); Writing – review & editing (equal). **Paul Bebbington:** Conceptualization (equal); Funding acquisition (equal); Investigation (equal); Methodology (equal); Supervision (equal); Writing – original draft (equal); Writing – review & editing (equal). **Philippa Garety:** Conceptualization (equal); Funding acquisition (equal); Investigation (equal); Methodology (equal); Project administration (equal); Supervision (equal); Visualization (equal); Writing – original draft (equal); Writing – review & editing (equal).

## Data Availability

The data sets generated and/or analysed during the current study will be available in anonymised form from the corresponding author (TW) on reasonable request, subject to review, following the publication of results.
